# Aerobic exercise combined with chlorogenic acid exerts neuroprotective effects and reverses cognitive decline in Alzheimer’s disease model mice (APP/PS1) via the SIRT1/ /PGC-1α/PPARγ signaling pathway

**DOI:** 10.3389/fnagi.2023.1269952

**Published:** 2023-11-16

**Authors:** Dan Shi, Zikang Hao, Wenxiao Qi, Fengyi Jiang, Kerui Liu, Xiao Shi

**Affiliations:** ^1^Yueyang Hospital of Integrated Traditional Chinese Medicine and Western Medicine, Shanghai University of Traditional Chinese Medicine, Shanghai, China; ^2^Academy of Integrative Medicine, Shanghai University of Traditional Chinese Medicine, Shanghai, China; ^3^Department of Physical Education, Ocean University of China, Qingdao, China; ^4^Sports Training College, Tianjin Institute of Physical Education, Tianjin, China; ^5^Faculty of Health Sciences, University of Macau, Taipa, China

**Keywords:** Alzheimer’s disease, physical activity, chlorogenic acid, oxidative stress, neuroinflammation, cognitive deficit

## Abstract

Alzheimer’s disease (AD) is a prevalent neurodegenerative disease account for 60–80% of the total number of people with dementia, but its treatment and prevention strategies are still in a long process of exploration. It has been reported that a healthy lifestyle may be an effective non-pharmacological intervention for the prevention and treatment of AD, including increased physical activity and the consumption of polyphenol-rich foods. This study, therefore, investigated the effects of 8 weeks of moderate-intensity aerobic exercise (EX), administration of chlorogenic acid administration (GCA), and a combination of both (EX+GCA) on β-amyloid (Aβ) deposition, inflammatory factors, oxidative stress markers, neuronal damage, and cognitive performance in the brains of AD model mice (APP/PS1) and which signaling pathways may be responsible for these effects. The study used Western blot to detect the expression of signaling pathway-related proteins, enzyme-linked immunosorbent assay to detect the expression of inflammatory factors, hematoxylin–eosin staining to detect hippocampal neuronal morphology, immunohistochemistry to detect changes in Aβ deposition in the hippocampus, an oxidative stress marker kit to detect oxidative stress status and the Morris water maze to detect changes in cognitive performance. This study showed that an 8-week intervention (EX/GCA/EX+GCA) activating the SIRT1/PGC-1α signaling pathway improved oxidative stress, neuroinflammation, Aβ deposition, and cognitive performance in mice. However, there was no obvious difference between the EX and GCA groups. In contrast, the combined EX+GCA intervention was significantly better than phase EX or GCA. Our study suggests that although relief of Aβ deposition, neuroinflammation, oxidative stress, neuronal damage, and cognitive decline could also be achieved with EX or GCA, the combined EX+GCA intervention showed better results. These relief effects on AD-related conditions may be obtained by mediating the activation of the SIRT1/PGC-1α signaling pathway. This study is the first to explore the improvement of AD-related conditions with a combined lifestyle of EX+GCA. This healthy lifestyle could be a candidate option for the treatment of AD.

## 1 Introduction

With advances in science and technology and improvements in healthcare, the average human life expectancy has generally increased, but this has also brought the problem of an aging population (Chang et al., 2019). The incidence of Alzheimer’s disease, which is closely linked to age, increases exponentially from age 65 onwards, with around 35 million people living with AD worldwide in 2009 alone (Scheltens et al., 2021). In addition, the financial pressure that AD brings to the patients themselves is also very significant. In the United States alone, the average cost of treating an AD patient is approximately US$174,000, and the annual cost of treating AD in the United States is as much as US$300 billion. Equally important is the cost of time and money to families caring for people with AD (Alzheimer’s Association, 2020).

Senile plaques formed by β-amyloid deposition and neurofibrillary tangles caused by aberrant phosphorylation of tau proteins are considered to be the main clinical features of AD. However, it is worth noting that, despite this, we still do not understand the relationship between the formation of tangles and the formation of plaques. The brain is resistant to amyloidosis for years or even decades. In addition, several other factors drive the course of AD, such as neuroinflammation, oxidative stress disorders, and aberrant programmed cell death (Ozben and Ozben, 2019; Bai et al., 2022; Bhatia et al., 2022). The search for ways to ameliorate these lesions is critical to preventing and mitigating AD.

Although the Food and Drug Administration (FDA) has approved several drugs for clinical use and researchers are actively developing new ones, although researchers are actively developing drugs for AD and the FDA has approved some as clinical options, they are not as effective as one might think, nor do they completely reverse the course of AD. Moreover, as mentioned earlier, the financial burden is not something that every AD patient and family would like to face, and therefore, the search for inexpensive interventions as an alternative means of reducing the risk of AD to some extent and slowing down the process of AD is attracting more and more attention from those involved (Bachurin et al., 2017). We must acknowledge the fact that, despite good results in animal and *in vitro* studies, some 450 therapies have failed in Phase II or Phase III clinical studies, with slightly more than half of these examples based on the “amyloid hypothesis” and its derivatives, and among the remaining therapies there are cases of failure based on other hypotheses as well (Knopman et al., 2021). Therapies such as *Aducanumab*, although capable of removing Aβ, have not been shown to slow or stop the progression of Alzheimer’s disease. In addition, the drug carries a risk of side effects; of the 1,029 patients in the 10 mg/kg dose group, 425 patients (41.3%) experienced problems with amyloid-related imaging abnormalities (ARIA), and 362 patients (35.2%) developed ARIA cerebral edema, with 94 of these experiencing associated symptoms such as headache, confusion, dizziness, and nausea. 197 patients (19.1%) developed ARIA microhemorrhages and 151 patients (14.7%) developed ARIA superficial iron deposits. Of these patients who experienced side effects, 14 were severe. Another drug targeting Aβ was *Lecanemab*, which delayed the worsening of the patient’s condition by about five months. In addition, patients who received *Lecanemab* were 31% less likely to progress to the next stage of the disease during the study period, but still did not achieve efficacy in reversing AD, and also experienced side effects similar to those of aducanumab - brain swelling and brain hemorrhage. For this reason, we should be cautious about developing therapies for Aβ and remain critical of the “amyloid hypothesis,” especially since it is increasingly recognized as the largest and longest-running biomedical failure in the field. Reversing AD may not be a breakthrough at this time, but delaying the process of AD is something we can focus on at this stage (Soderberg et al., 2023). The search for effective and inexpensive alternative therapies has, therefore, become a hot topic of interest. In recent studies, it has been suggested that healthy lifestyles such as physical activity (Sujkowski et al., 2022) and dietary supplements (McGrattan et al., 2019) can be effective in preventing and alleviating AD. In clinical human studies, it has been found that long-term moderate exercise can improve the cognitive decline caused by AD and increase the ability to perform daily living activities (Dauwan et al., 2021). In addition to this, studies in AD-related animal models have found that low to moderate-intensity aerobic training not only promotes the clearance of Aβ deposits in the hippocampus but also reverses hippocampal neuroinflammation, blood–brain barrier destabilization and neuronal apoptosis caused by AD (Zhang et al., 2019; Chen et al., 2020). Long-term aerobic exercise also promotes the regeneration of hippocampal neurons, and LTP, which is closely related to synaptic plasticity, is also beneficially improved by exercise (Dao et al., 2015, 2016). In addition to exercise, the addition of polyphenol-rich foods to the daily diet may also have a role in the prevention and relief of AD (Amato et al., 2019), and one such substance, chlorogenic acid, is of broad interest for its powerful antioxidant, anti-inflammatory effects. Chlorogenic acid is not only present in our daily diet in the form of coffee or green coffee but is also found in many vegetable juices, teas, and other everyday products and can be said to be closely linked to people’s lives (Santana-Galvez et al., 2017). In an *in vitro* study, GCA was found to protect neuronal cell activity by reducing the accumulation of ROS, inhibiting BACE1 and β-secretase activity, and thereby protecting neuronal cells from oxidative stress and apoptosis. In an animal model of AD, GCA treatment reversed Aβ deposition and disruption of synaptic plasticity due to AD and protected hippocampal neuronal cells from neurotoxic damage, in addition to being associated with inhibition of AChE and BChE activity (Lee et al., 2011; Pavlikova, 2023). In addition to several meta-analyses showing a significant reduction in the risk of AD in long-term coffee drinkers compared to non-coffee drinkers, prospective studies have analyzed chlorogenic acid intake as perhaps protective against AD, which strengthens the case for protecting the brain from the risk of AD through chlorogenic acid (Butt and Sultan, 2011). Taken together, these studies provide strong evidence for the beneficial effects of EX and GCA in the prevention and treatment of AD.

Recent reports have shown that resveratrol, which is a polyphenol together with GCA, exerts superior neuroprotective, Aβ toxicity reducing and apoptosis inhibiting effects when combined with EX than when resveratrol is taken alone or when EX is performed (Broderick et al., 2020). Based on this, it is reasonable to assume that EX and GCA may have a mutually reinforcing effect in terms of neuroprotection and AD treatment, i.e., the combination of the two may exert a better effect than EX or GCA alone. Therefore, the objectives of the present study were to explore whether EX combined with GCA supplementation showed superior effects on the clearance of Aβ deposition, alleviation of oxidative stress state, reversal of cognitive decline, and clearance of inflammatory factors in the hippocampus of AD mice compared to EX or GCA alone, and of course to explore through which signaling pathways they may exert these effects, which may provide future insights into the prevention and improvement, which may provide a new option. Although EX/GCA have shown beneficial effects in cellular, animal, or human studies and represent two different complementary therapies related to AD protection (polyphenols/exercise therapy), to our knowledge, previous studies have not explored the combined effects of the two, which have shown better results in some other chronic diseases. Whereas resveratrol, which is also a polyphenol, and curcumin have been studied in combination to intervene in AD and also show better results; based on this, we hypothesize that EX in combination with GCA will have the same effect. While previous studies have suggested that exercise or polyphenols may mediate the activation of SIRT1 to exert a protective effect against AD and that its downstream targets play an important role in inhibiting some of the critical markers of AD, we also hypothesized that the combination of the two would also activate SIRT1 and that it would exert a better effect than its use alone (Springer and Moco, 2019; Zia et al., 2021).

## 2 Materials and methods

### 2.1 Animals

Animals for this study were taken from wild-type C57BL/6 mice as a blank control group and double transgenic mice heterozygous for APP and PSEN1 on a C57BL/6 background (APP/PS1) as a model group. The animals used were purchased from the Institute of Model Zoology, Nanjing University, and the animal certificate number is (201400975). Seventy-five mice, 6 months old, were purchased. The study was divided into five groups: wild type C57BL/6 as the blank control group (CON); AD control group; (AD) chlorogenic acid + AD group (GCA + AD); aerobic exercise + AD group (EX+AD) and chlorogenic acid + aerobic exercise + AD group (GCA + AD+ EX), each group contained 15 mice. The mice were housed in 5 to a cage in standard rearing cages or in single cages in case of fighting. Housing conditions were 20–25°C with relative humidity maintained at 50–55%, ensuring adequate standard maintenance chow and water, *ad libitum* ingestion, avoidance of bright light and noise, and a 12/12 h cycle of dark/light time. The study was approved by the Laboratory Animal Ethics Committee of Shanghai University of Traditional Chinese Medicine (ethics number: PZSHUTCM210702001) on the basis of the guidelines for the use of laboratory animals in the People’s Republic of China and the “3Rs” principle was followed during the experiments (Aske and Waugh, 2017). All animals were acclimatized for 1 week after arrival in the laboratory before the rest of the treatment (Buchanan et al., 2012).

### 2.2 Chlorogenic acid (GCA) intervention

GCA was purchased from Maclean’s (product no. C805057) at a minimum purity of 98%. The dose of GCA was selected based on the results of previous studies and was administered by intraperitoneal injection of GCA dissolved in 0.9% saline at 80 mg/kg/d per mouse receiving the GCA intervention (Oboh et al., 2013; Fukutomi et al., 2021), while the other groups not receiving GCA were injected with the same volume of 0.9% saline. GCA was administered daily at 9 a.m. for 8 weeks.

### 2.3 Exercise

The animal experimental treadmill table used in this study was purchased from Beijing Smart Mouse Dobao Biotechnology Co., Ltd. (product number: DB030X), and the exercise scheme was developed by summarizing the experience of previous studies and making minor modifications to simulate the intensity of moderate-intensity aerobic exercise (Zhang et al., 2019). Briefly, before the formal treadmill training, the mice requiring exercise treatment were given a week of acclimatization training to learn the use of the treadmill (Day 1: 5 m/min, 30 min; Day 3 and Day 4: 8 m/min, 30 min; Day 5–7: 12 m/min, 30 min) for a total of 5 days. At the end of the acclimatization period, the formal treadmill training program begins: (warm-up: 6 m/min, 5 min; training: 9 m/min, 5 min + 13 m/min, 28 min; cool-down: 6 m/min, 4 min), 5 days per week, total 9 weeks of acclimatization + formal training period. Each exercise session starts at 17:00 daily, depending on the rodent’s habits. The use of moderate intensity was developed by combining human and animal studies. Firstly, the vast majority of AD patients are elderly patients whose physical functions, such as cardiorespiratory capacity and bone toughness, may not be able to withstand intense strenuous exercise, which may bring them sports injuries. Secondly, according to the viewpoints of some existing researchers, excessive high-intensity exercise may cause the secretion of pro-inflammatory factors by the skeletal muscles, resulting in acute inflammation. While according to the latest research, inflammation is the most significant disadvantageous factor of AD, and the purpose of exercise intervention is to inhibit or remove these inflammatory factors. High-intensity exercise may lead to putting the cart before the horse in terms of the purpose of the intervention. Lastly, the exercise protocol chosen in this study is the one that simulates human moderate-intensity exercise (55–70% HRmax), which has been commonly used in related research in this field (Voss et al., 2013; Springer and Moco, 2019).

### 2.4 Morris water maze

The Morris water maze test was chosen for this study to assess changes in the cognitive and spatial learning memory abilities of mice (Vorhees and Williams, 2014). The detailed methodology was reported in a previous study (Zhang et al., 2023). Briefly, the experiment was conducted in a 1.5 m diameter, 0.3 m deep constant temperature pool (water temperature constant 22°C), divided into four quadrants, in one of which a 9 cm diameter hidden platform was placed and the water in the pool was rendered opaque by fuel immersion. A video camera was placed directly above the center of the pool for recording purposes.

**Figure 1:**
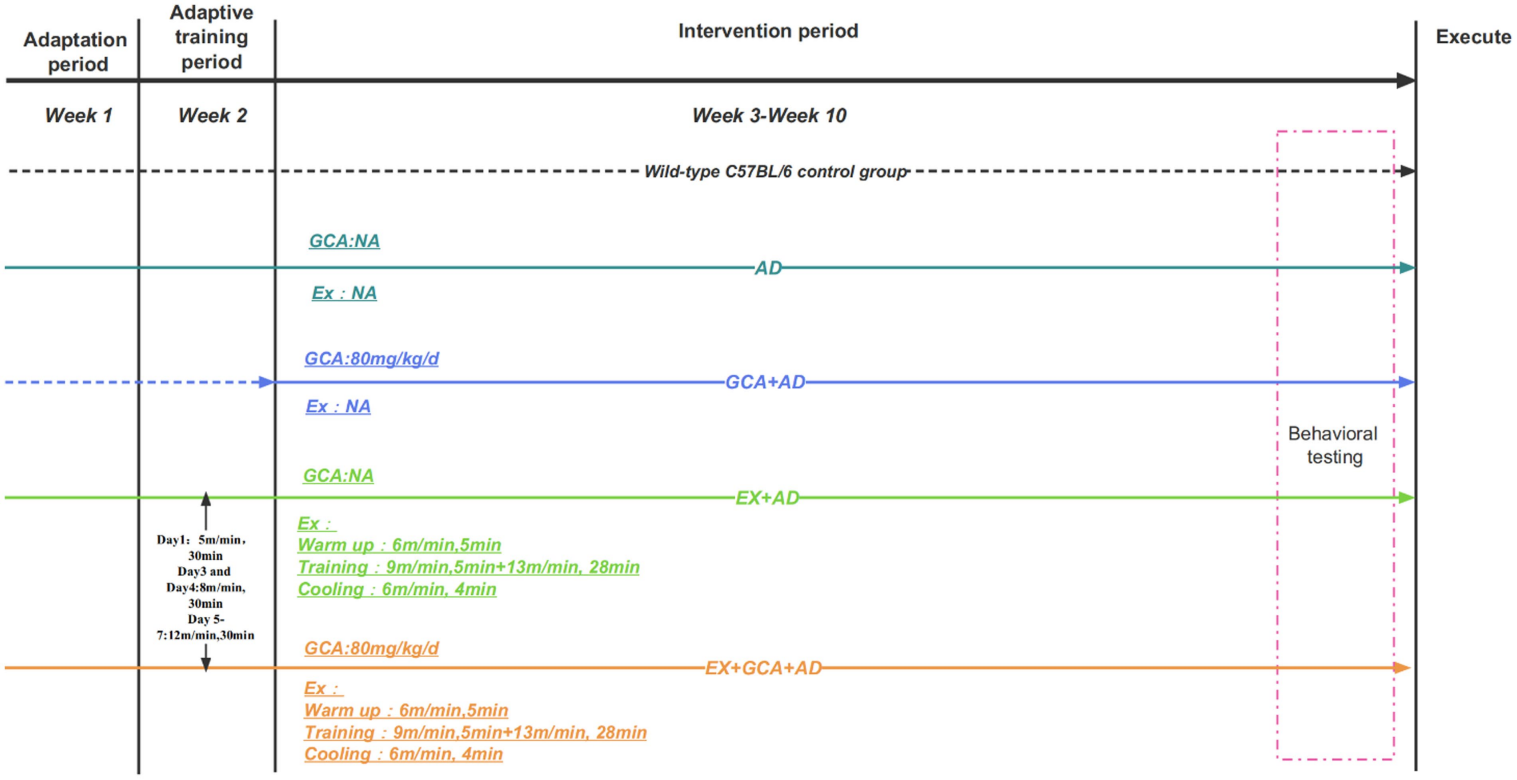
Outline of the experimental design process. The dotted line in the graph represents non-participation in treadmill exercise. NA, Representatives do not participate in this intervention.

The experiment was conducted over 6 days, with mice being trained to find the platform within 1 min on the first 5 days, and if they still could not find the platform after more than 1 min, they were guided to the platform and held on it for 15 s. Each mouse was trained three times a day at 15-min intervals, and on the sixth day, the platform was removed, and the spatial exploration experiment was recorded. The entire intervention process is shown in Figure 1.

### 2.5 Tissue preparation

The day after the water maze test was performed, all mice were euthanized immediately after pentobarbital overdose administration, brain tissue was removed, and hippocampal bodies were isolated on ice. Hippocampal tissue was randomly selected from a number of mice (*n* = 3) in each group, and the left brain was analyzed for the distribution and area of Aβ plaques within the hippocampus of the mice using immunohistochemical techniques; the right brain was analyzed for pathological changes in the hippocampal tissue of the mice using HE staining. Three of the remaining mouse hippocampal samples were then selected for freezing and stored in a-80°C refrigerator for subsequent WB analysis. A further three samples were randomly selected for ELISA analysis. The final remaining samples (*n* = 6) were also frozen in a-80°C freezer for oxidative stress detection. Here, the mice selected for our study regarding each experimental method were in their respective groups and were randomly selected; the reason for the selection of three was to ensure that three sets of replications could be made for each outcome to ensure the accuracy of the study. During the grouping phase of the study, the four groups of APP/PS1 mice and wild control mice were selected randomly, and the intervention implementer and study designer for the study were not the same person, i.e., the intervention implementer was not aware of exactly which groups of mice were involved. All experimental methods were subjected to three repeated measurements, and specific raw data can be found in the Supplementary Tables.

### 2.6 Hematoxylin–eosin staining

The extracted tissues were quickly dehydrated and transparent, embedded in wax, and then made into wax blocks. The wax blocks were cut into 4 mm thick sections using a slicer; the sections were dewaxed and then subjected to HE staining. Briefly, the sections were stained in hematoxylin aqueous solution (1 g hematoxylin +15 g potassium aluminum sulfate +10 mL anhydrous ethanol +100 mL distilled water) for 4 min, then after 20 s of ammonia separation, they were placed in eosin staining solution (eosin 1 g + 100 mL 90% alcohol) for 2 min, then the stained sections were dehydrated in pure alcohol, then xylene was used to make the sections transparent, and finally they were sealed with Neutral gum was used to seal the sections.

### 2.7 Immunohistochemistry

Immunohistochemical techniques were used to detect Aβ plaques in mouse hippocampal tissue according to the methods of previous studies (Lu et al., 2017). Briefly, sections were made of paraffin blocks that had been prepared and placed in 90, 80, and 70% concentrations of alcohol, followed by distilled water. The sections were removed from the wipe, and peroxidase blocker (0.3% hydrogen peroxide) was added dropwise and incubated for 15 min at room temperature for 3 h. The sections were rinsed three times with PBS buffer for 5 min each. The sections were then stained with DAB and hematoxylin for 8 min each and finally dehydrated in graded alcohol solution, cleared with xylene, and covered with neutral resin. The stains were placed under a microscope for observation (Clavaguera et al., 2013).

### 2.8 Western blot

Seahorse tissue was ground and broken up in liquid nitrogen, placed in a homogenizer and lysis buffer [150 mM NaCl, 1.0% NP-40,0.5 sodium deoxycholate, 0.1% SDS (sodium dodecyl sulfate), 50 mM Tris, pH 8.0, Abcam] was added and samples were lysed for 30 min in 1.5 mL centrifuge tubes at 4°C The supernatant was stored in a refrigerator at −20°C. The sample was centrifuged at 12000 rpm for 5 min. Using the BCA kit, protein quantification was performed on the samples to be tested. Different concentrations of separation gel (DD H2O, 30% Acrlamide, 1.5 M Tris–HCL, pH 8.8,10% SDS, 10% g ammonium persulfate, TEMED) and concentrated gel were configured according to the molecular weight of the proteins to be measured, and the proteins were separated at a constant pressure of 100v in the electrophoresis solution, followed by transfer of the proteins to PVDF membranes in the transfer solution. The primary antibody working solution was configured using a 5% milk-blocking solution for 1 h. The primary antibody was incubated overnight at 4°C and washed using TBST solution for 10 min × 3 times. The secondary antibody working solution was added dropwise to PVDF and incubated for 1 h at room temperature, and the above steps were repeated to wash the secondary antibody. Finally, the proteins to be tested were visualized by the luminescence imaging system using ECL reagents (Pillai-Kastoori et al., 2020).

### 2.9 Enzyme-linked immunosorbent assay

The levels of IL-1β, IL-6, and TNF-α were measured in mouse hippocampal tissue homogenates according to the ELISA kit manufacturer’s instructions (RK00006; RK00008; RK00027, ABclonal Biotechnology Co., Ltd., Wuhan, China) and the OD values of each group of samples were measured in a spectrometer and calculated (Andreasson et al., 2015).

### 2.10 Oxidative stress testing

ROS, SOD, MDA, CAT, GSH-PX, and H_2_O_2_ were measured using kits from Abbkine Technology Ltd. (Wuhan, China) according to the manufacturer’s recommendations.

### 2.11 Data analysis

Data from this study were analyzed using Graphpad Prism 9.5 (Graphpad Software, Boston, USA), and the resulting data were first subjected to a Shapiro–Wilk test to check for normal distribution, followed by a one-way ANOVA to detect between-group differences, and finally *post hoc* Tukey’s multiple comparisons (Tseng et al., 2023). For WB experiments and immunohistochemical studies, the grey-scale values of protein bands and the number and area of Aβ plaques were analyzed using ImageJ software (Ertekin et al., 2016).

## 3 Results

### 3.1 EX/GCA/EX+GCA alleviates cognitive decline in AD mice

**Figure 2:**
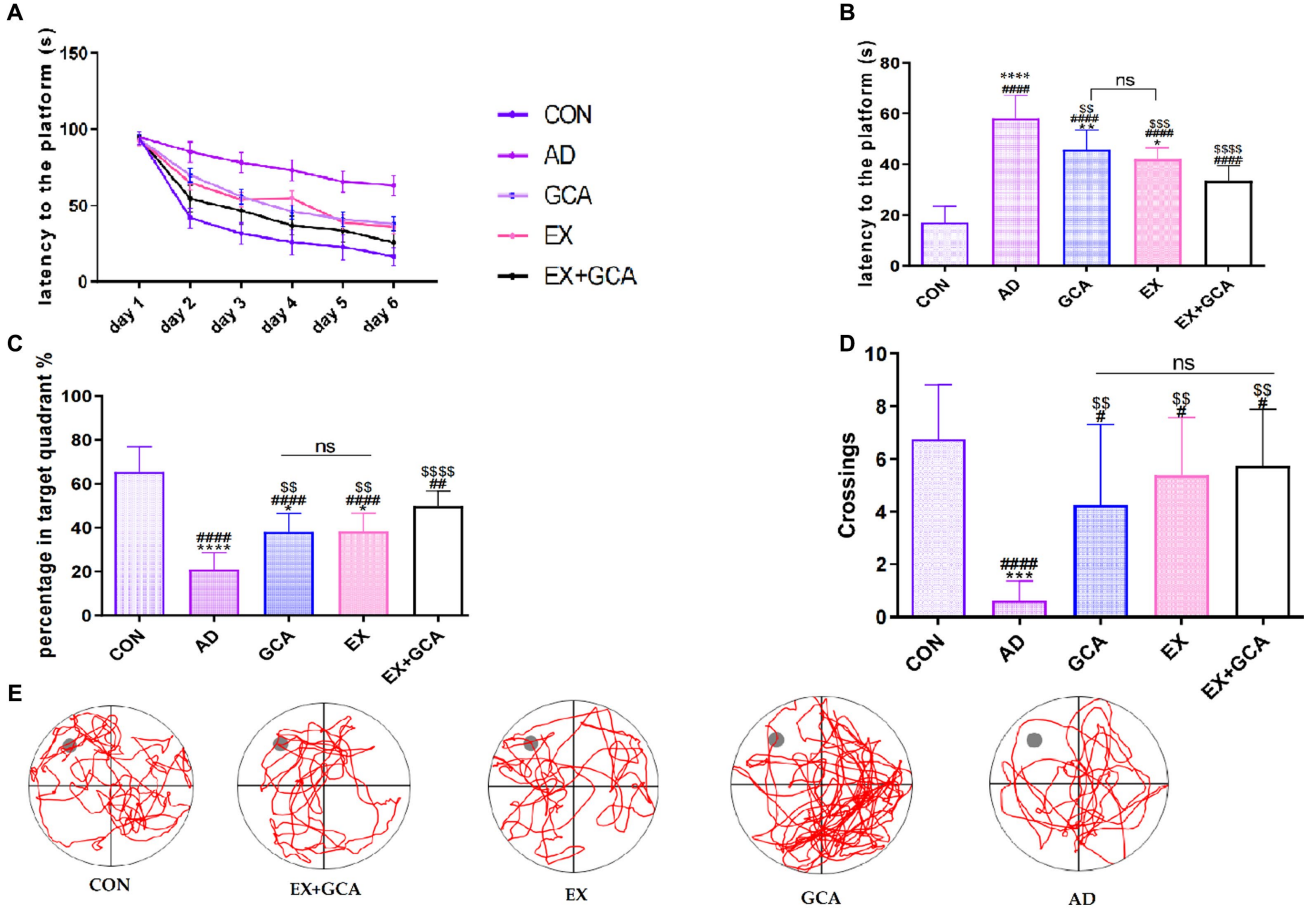
Results of the MWM test in mice. **(A)** Learning memory curve of mice; **(B)** mean time to find the platform at the last training session; **(C)** percentage of time spent in the quadrant where the platform was located as a percentage of total escape time; **(D)** number of times mice crossed the location of the platform; **(E)** lane trajectory; CON, wild-type C57BL/6 blank control mice; AD, APP/PS1-mounted AD mice receiving no intervention; GCA, APP/PS1-mounted AD mice treated with chlorogenic acid; EX, APP/PS1-mounted AD mice treated with moderate intensity aerobic exercise; EX+GCA, APP/PS1-mounted AD mice treated with both chlorogenic acid and moderate intensity aerobic exercise. ns: no significant difference; #/##/###/####: CON vs. the other four experimental groups, *p* < 0.05/0.01/0.001/0.0001; */**/***/****: in all AD experimental groups, *p* < 0.05/0.01/0.001/0.0001 vs. EX+GCA; $/$$/$$$/$$$$: *p* < 0.05/0.01/0.001/0.0001 vs. AD in all AD experimental groups. The symbols in all the remaining charts in this document have the same meaning as here.

Decreased learning ability and higher cognitive abilities are considered to be an essential clinical condition in AD; therefore, the MWM test was used in this study to assess the effect of AD on cognitive abilities and the effect of exercise or chlorogenic acid administration or exercise combined with chlorogenic acid on the alleviation of cognitive decline induced by AD. As shown in Figures 2A,B, the escape latency was significantly longer in the transgenic AD mice compared to the wild control group (*p* < 0.05), indicating that AD impaired cognitive performance in the mice. However, among all AD mice, those receiving treatment with GCA/EX/GCA + EX had a significantly lower escape latency than those not receiving treatment (*p* < 0.05), but on the last day of testing, it was found that escape latency was not significantly different between the GCA and EX groups, whereas AD mice receiving both GCA and EX showed a significantly lower escape latency than those receiving treatment alone The AD mice receiving both GCA and EX showed a significant decrease in escape latency (*p* < 0.05). As shown in Figures 2C,D, on the last day of training, the platform was removed, and the percentage of time the mice spent crossing the platform and staying in the third quadrant was recorded. It was found that the mice in the CON group crossed the platform significantly more often and stayed for a longer percentage of time than the rest of the groups. In the AD mice, it was found that mice receiving both GCA and EX treatment had significantly more limited (*p* < 0.05) than those receiving GCA or EX treatment alone. The above results suggest that AD mice exhibit a significant decrease in cognitive and spatial memory abilities relative to healthy mice, while the cognitive abilities of mice receiving GCA or EX were rescued, and this effect was more pronounced after the combined treatment of GCA + EX.

### 3.2 EX/GCA/EX+GCA alleviates the overexpression of inflammatory factors in the hippocampus of AD mice

**Figure 3:**
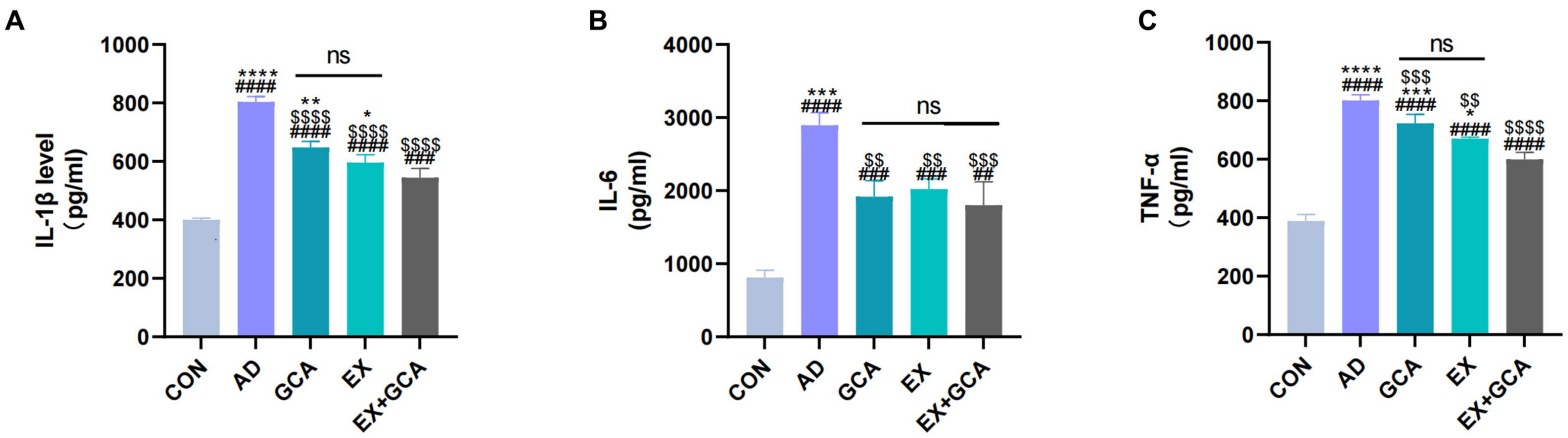
Expression of inflammatory factors in mouse hippocampal tissues. **(A)** Expression status of IL-1β in mouse hippocampal tissues; **(B)** Expression status of IL-6 in mouse hippocampal tissues; **(C)** Expression status of TNF-α in mouse hippocampal tissues. ns: no significant difference; #/##/###/####: CON vs. the other four experimental groups, *p* < 0.05/0.01/0.001/0.0001; */**/***/****: in all AD experimental groups, *p* < 0.05/0.01/0.001/0.0001 vs. EX+GCA; $/$$/$$$/$$$$: *p* < 0.05/0.01/0.001/0.0001 vs. AD in all AD experimental groups.

Based on recent studies suggesting that neuroinflammation is often found in the AD brain and may be a cause of exacerbation of AD, the present study examined inflammatory factors (IL-1β, IL-6, TNF-α) in mouse hippocampal tissue homogenates using the ELISA technique. As shown in Figure 3, inflammatory factors were significantly increased in the hippocampal homogenates of mice in the transgenic AD group relative to the CON group (*p* < 0.05). The expression of IL-1β, IL-6, and TNF-α was significantly decreased in AD mice treated with GCA or EX or EX+GCA relative to untreated AD mice, and in these treated mice, it was also found that in the hippocampal tissue of AD mice treated with both GCA and EX relative to AD mice treated with only one treatment modality, the expression of IL-1β and TNF- α expression was significantly reduced. Interestingly, with regard to IL-6 expression, there was no significant difference between the three treatment modalities, although all three groups of AD mice receiving treatment showed a decreasing trend. In summary, inflammatory factor overexpression did exist in AD mice but was reversed in the AD brain after treatment, suggesting that, alone or in combination with GCA or EX treatment, it could alleviate chronic neuroinflammation in the AD brain to some extent.

### 3.3 EX/GCA/EX+GCA alleviates oxidative stress in AD mice

**Figure 4:**
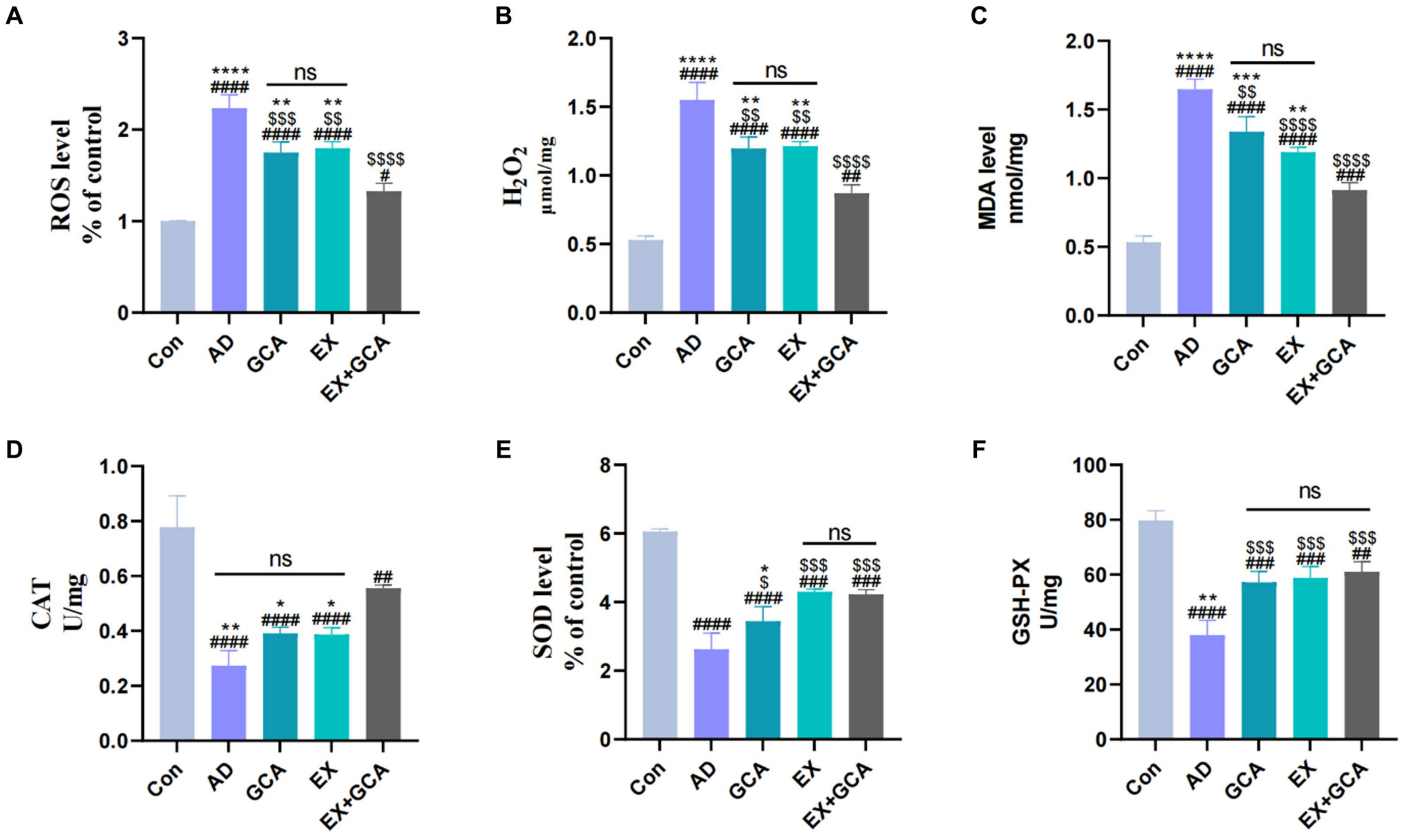
Changes in markers of oxidative stress in mice. **(A)** reactive oxygen species (ROS); **(B)** hydrogen peroxide (H2O2); **(C)** malondialdehyde (MDA); **(D)** catalase (CAT); **(E)** superoxide dismutase (SOD); **(F)** glutathione peroxidase (GSH-PX).

Previous studies have suggested that AD may be associated with a disruption of the oxidative/antioxidant balance and that oxidative stress may exacerbate the process of AD. In this study, three representative oxidative and antioxidant stress indicators were selected to assess the oxidative stress status in AD and after treatment with GCA or EX or EX+GCA. H_2_O_2_, a product of reactive oxygen metabolism, is often involved in the process of neurodegenerative disease together with NF-kB (Hadipour et al., 2020), and in this study, it was found that H_2_O_2_ levels were distinctly increased in AD mice compared to the CON group, while in H_2_O_2_ decreased distinctly after receiving treatment and was best in GCA + EX, but there was no significant difference between the two groups, EX and GCA. In addition, according to reports, increased ROS may cause ferroptosis and pyroptosis in microglia and neurons, a pro-inflammatory type of programmed cell death, so this study also examined changes in ROS (da Silva and Ming, 2007), which were consistent with changes in H_2_O_2_, confirming that the amount of ROS determines the amount of H_2_O_2_. In addition to this, the study also examined the changing status of MDA (Bermejo et al., 1997), where oxygen free radicals act on the unsaturated fatty acids of lipids to produce lipid peroxidation; the latter is gradually broken down into a complex series of compounds, including MDA. Lipid peroxidation is thought to be closely related to AD, and in this study, it was found that MDA levels were distinctly increased in AD mice compared to the CON group, while after treatment, MDA decreased significantly and GCA + EX had the best effect, although there was no significant difference between GCA and EX, compared to GCA + EX. SOD is an essential antioxidant against superoxide radical toxicity in all cells exposed to O2 (Farajdokht et al., 2017), and in this study, AD mice showed a significant decrease in SOD compared to the CON group and a significant increase in MDA after treatment, although there was no significant difference between EX and EX+GCA. Protection against oxidative damage to cells was provided by the removal of H2O2 from cells using catalase, which was found to significantly reduce CAT levels in AD mice compared to the CON group, but after treatment, only EX+GCA showed a significant difference relative to the AD group. Finally, this study analyzed GSH-PX (Zhi-bin et al., 2010), which was significantly reduced in the AD mice compared to the CON group and, after treatment, was significantly elevated in all three groups, but there was no significant difference between the three treatments. In summary, it can be found that in AD, a state of oxidative stress does exist, but after GCA/EX/EX+GCA, the oxidative stress is alleviated. As shown in Figure 4.

### 3.4 EX/GCA/EX+GCA alleviates neuronal damage and reduces Aβ deposition in AD mice

**Figure 5:**
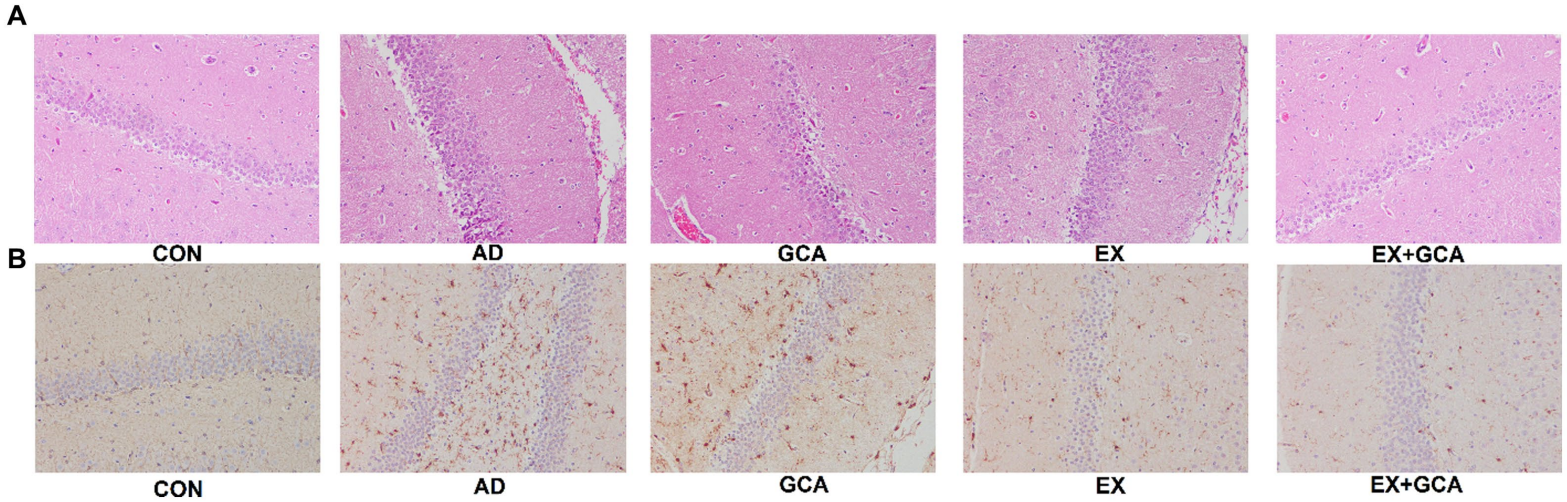
Neuronal damage and Aβ deposition in the hippocampus of mice. **(A)** HE staining of hippocampal tissue, **(B)** Immunohistochemistry of hippocampal tissue.

After observation by HE staining, we found that compared with the mice in the CON group, the rest of the groups showed different degrees of neuronal loss or reduction, and the hippocampal tissues became more disorganized and loose, whereas after treatment with EX/GCA/EX+GCA, the degree of neuronal loss or reduction was mitigated, and the hippocampal tissues showed a more regular arrangement compared with those of the AD mice without the intervention, with the EX+GCA having a more favorable effect. As shown in Figure 5A.

Aβ deposition in the hippocampus of mice was exacerbated by AD, but EX+GCA significantly alleviated the worsening of Aβ deposition, as shown in Figure 5B.

### 3.5 EX/GCA/EX+GCA activates the SIRT1/PGC-1α signaling pathway

**Figure 6:**
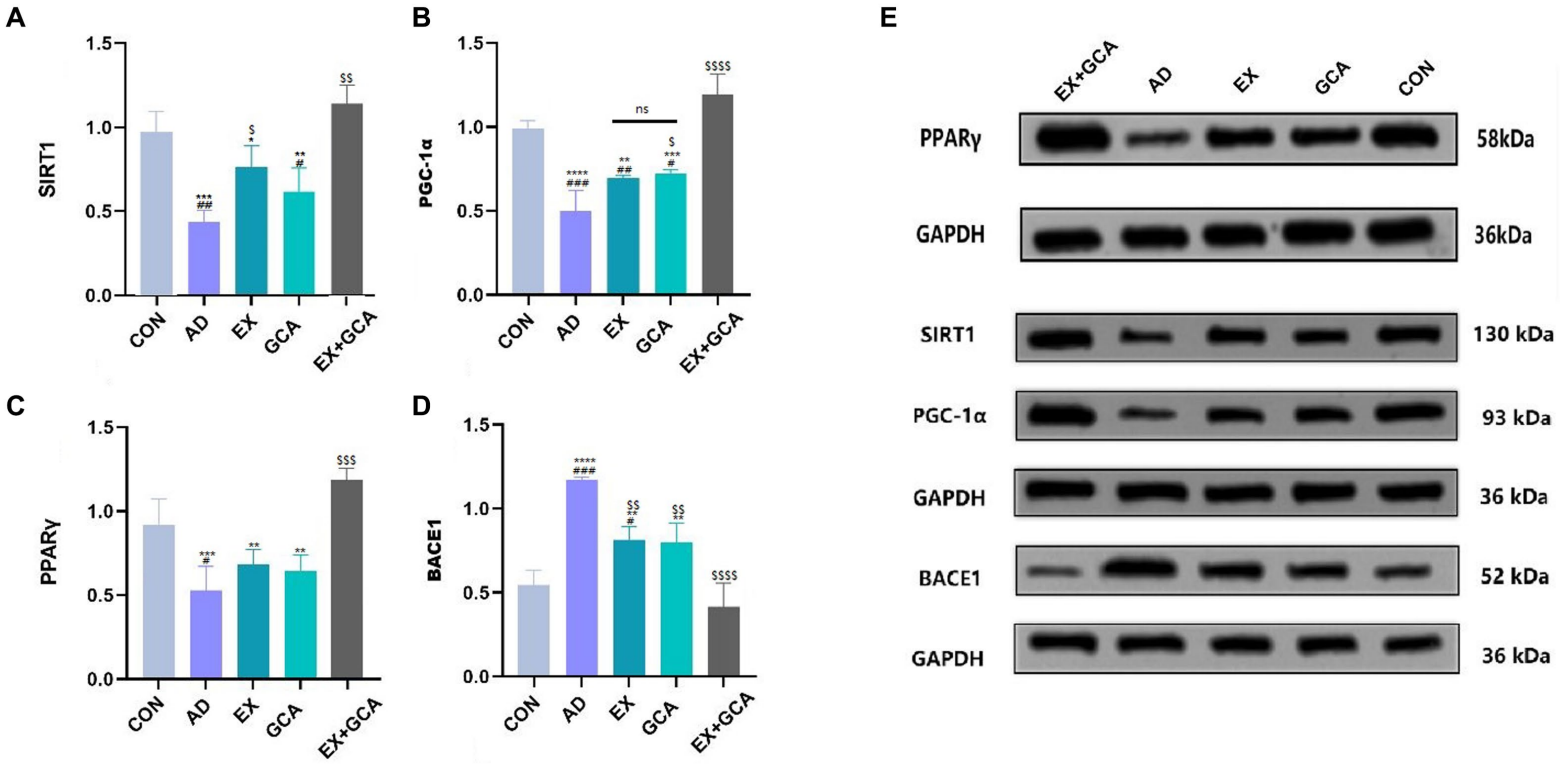
Activation or inhibition of relevant signalling pathways in mouse hippocampal tissue. **(A)** SIRT1; **(B)** PGC1-α; **(C)** PPARγ; **(D)** BACE1; **(E)** Immunoblot image of SIRT1/PGC-1α/PPARγ/BACE1.ns, no significant difference; #/##/###/####: CON vs. the other four experimental groups, *p* < 0.05/0.01/0.001/0.0001; */**/***/****: in all AD experimental groups, *p* < 0.05/0.01/0.001/0.0001 vs. EX+GCA; $/$$/$$$/$$$$, *p* < 0.05/0.01/0.001/0.0001 vs. AD in all AD experimental groups.

Sirtuins are nicotinamide adenine dinucleotide (NAD +)-dependent deacetylases classically associated with calorie restriction and aging in mammals. Of the seven family members, SIRT1 plays a vital role in the treatment of neurodegenerative diseases and is often considered a therapeutic target due to its positive correlation with the structural integrity of neurons in the brain, the ability to learn and to remember, and the possibility that SIRT1 may also have a significant link to the APP hydrolysis pathway in AD (Herskovits and Guarente, 2014). In this study, SIRT1 expression was inhibited in the hippocampus of untreated AD mice, followed by inhibition of PGC-1α and consequent inhibition of PPARγ, a downstream factor regulated by PGC-1α, which was followed by upregulation of BACE1 expression. In contrast, EX/GCA/EX+GCA reactivated SIRT1, PGC-1α, and PPARγ, which were inhibited by AD, and subsequently BACE1 expression was inhibited. As shown in Figure 6.

## 4 Discussion

Healthylifestyles can prevent and improve neurodegenerative diseases, including AD, which is closely related to age, and among healthy lifestyles, physical activity, and diet seem to be highlighted (Pardo-Moreno et al., 2022). Regular and long-term exercise and the consumption of polyphenol-rich foods appear to be associated with better patient compliance and side effects than medication. According to previous studies, these two healthy lifestyles have been shown to have considerable effects on improving cerebral blood flow in AD, maintaining blood–brain barrier stability, reducing chronic neuroinflammation, and increasing synaptic plasticity (De la Rosa et al., 2020; Bukhari, 2022; Caruso et al., 2022). This study examined the effects of three healthy lifestyle-related measures on AD, and after 8 weeks of intervention, we found that EX/GCA/EX+GCA all had beneficial effects on (1) hippocampal Aβ deposition, (2) hippocampal neuroinflammation, (3) oxidative stress (4) cognitive performance and partially reversed damage due to AD, possibly through SIRT1/ PGC-1α/BACE1 signaling pathway. On this basis, the study found that moderate-intensity aerobic exercise in the form of a treadmill combined with chlorogenic acid supplementation at 80 mg/kg daily showed additional protective effects over EX and GCA alone. There appears to be a mutually reinforcing effect between the two.

APP/PS1 double transgenic mice are mice transfected with both human APP and PS1 exogenous mutant genes. This type of mouse has a faster rate of Aβ production and earlier age-related plaque formation, and this model is an internationally recognized animal model for studying AD-related conditions (Dawson et al., 2018). In this study, in addition to using APP/PS1 mice, we added wild background mice of APP/PS1 mice, i.e., C57BL/6 mice, as a blank control group. At the end of the entire intervention cycle, it was found that the remaining four groups of APP/PS1 mice all showed significantly different AD-related lesions relative to the wild background mice, but in all four groups because of the presence of different modalities of the extent of change in these lesions was also significantly different in these four groups due to the presence of different treatment modalities.

Cognitive decline is the main clinical symptom of AD, and the hippocampus is the central area of the body where learning and memory are accomplished (Hugo and Ganguli, 2014). The study found that escape latency and total distance traveled decreased during the experiment for both groups, with the wild control mice taking the shortest time and traveling the shortest distance and the AD mice taking a higher time than the wild control mice. The quadrant dwell time and number of crossings were better in the wild control group than in the experimental group, which is consistent with previous studies (Wang X. et al., 2018; Ishida et al., 2020; Ryu et al., 2020; Web of Science, 2021; Lu et al., 2022; Peng et al., 2022), and we found that after EX/GCA/EX+GCA treatment, both memory and cognitive abilities were effectively alleviated in the mice, which is thought to be because the 8-week treatment delayed the progression of AD and effectively inhibited cognitive decline.

Both in animal models and in autopsies of AD patients, a large number of senile plaques are found in the hippocampus, and the main component of senile plaques is Aβ deposition (DeTure and Dickson, 2019). Under normal conditions, Aβ production and clearance are in a dynamic equilibrium, but under abnormal transitions, β and γ-secretase activity increases, and excessive shifts in the hydrolytic pathway of APP to the β and γ-secretase pathways result in excessive Aβ deposition (Chen et al., 2017; Ashrafian et al., 2021). Previous studies have shown that Aβ deposition begins to occur in APP/PS1 mice at 3–4 months of age, and significant plaque production is observed around 6 months of age (Shi et al., 2017); the same phenomenon was found in the present study, with mice in the AD group showing varying degrees and sizes of Aβ plaques compared to wild controls, but the number and size of plaques in the EX/GCA/EX+GCA group were smaller than in the AD group due to treatment. Previous studies have reported on the effect of EX or GCA on the clearance of Aβ deposits. Zhang et al. (2019) found that after 12 weeks of treadmill intervention, Aβ deposits were effectively cleared from the hippocampus of APP/PS1 mice, and in addition to this, a decrease in the number of activated microglia was observed. The effect of GCA on the clearance of Aβ was reported several years ago, with the breakdown of Aβ deposits and protofibrillar Aβ species into Aβ peptides in AD mice after 50 mg/kg/d chlorogenic acid intervention, in addition to the neuroprotective effect of GCA and its derivatives in counteracting the aggregation of Aβ42 in SH-SY5Y cells in *in vitro* studies (Gao et al., 2021). However, it is of concern that the clearance of Aβ by GCA may be dose-dependent, so this study combined with previous studies and chose a dose below the maximum dose of 80 mg/kg/d for the intervention. The dose of chlorogenic acid selected for this study was chosen based on previously reported studies and contains two aspects: potency and toxicity. According to the study, although high doses of chlorogenic acid will have a better effect initially, there is a bottleneck effect, i.e., the potency will not continue to increase after it rises to a certain level, but instead, it will decrease to an effect equal to that of a smaller dose, in addition, a higher dose may also. In addition, higher doses may also cause hepatotoxicity, which must be taken into account, so in this study a dose of 80 mg/kg/day was chosen. In addition, according to the results of human studies, the intestinal absorption of chlorogenic acid is not excellent; only about 1/3 will be absorbed by the intestinal tract, so all things considered, the dose of 80 mg/kg/day of chlorogenic acid can better simulate the absorption effect of the human body and has the highest potency ratio (Wang Z. et al., 2018; Caruso et al., 2022). While single polyphenols have been observed to be effective in animal experiments or cohort studies, these results cannot be replicated in human trials, so we should focus on a variety of foods with high polyphenol content when advancing our research to the clinical stage rather than discussing the effects of a single polyphenol alone, and it is true that we consume foods that are rich in polyphenol species when they are abundant, rather than a single one (Rao et al., 2012, 2023).

In addition, the present study found that BACE1 expression was significantly upregulated in the hippocampus of AD mice compared to wild control mice; BACE1 is often considered to be a β-secreting enzyme, although BACE1 also exists as a homolog of BACE2, BACE1 is mainly distributed in neurons of the central nervous system (CNS) while BACE2 is mainly distributed in peripheral tissues, BACE1 transcript levels are often positively correlated with Aβ, and therefore inhibition of BACE1 expression may inhibit Aβ production and deposition (Yan and Vassar, 2014; Shah et al., 2018; Hampel et al., 2021). In our study, we found that inhibition of BACE1 expression was observed regardless of the treatment, which is consistent with previous studies. In addition, we examined the expression of PGC-1α, an upstream factor that regulates BACE1 expression, which was upregulated in response to exercise or chlorogenic acid. Interestingly, in addition to PGC-1α, SIRT1 expression was reported to be negatively correlated with BACE1, with SIRT1 overexpression and subsequent downregulation of BACE1 expression in response to exercise or chlorogenic acid being observed in both *in vitro* and AD models (Marwarha et al., 2014), and Aβ deposition being alleviated. In addition to this, it has also been suggested that SIRT1 may regulate PGC-1α to achieve BACE1 inhibition due to the acetylation of SIRT1 at the lys268,293 site tor PPARγ, thereby increasing the activity of PGC-1α, which continues to act directly on the peroxisome proliferator response element at the BACE1 promoter to inhibit BACE1 activity, which was further confirmed in our study (Herskovits and Guarente, 2014; Wong and Tang, 2016; Ye and Wu, 2021). It is, therefore, reasonable to assume that EX/GCA/EX+GCA is likely to inhibit Aβ expression via the SIRT1/PGC-1α pathway, but it is well worth noting that the combination of EX and GCA is more effective for activation of this pathway and more effective for inhibition of BACE1.

Furthermore, according to previous reports, the presence of persistent and unrelieved neuroinflammation in the AD brain exacerbates the course of AD, and the long-term accumulation of unresolved inflammatory factors may continue to exacerbate neuroinflammation and neuronal degeneration by causing oxidative stress damage (Calsolaro and Edison, 2016; Xie et al., 2022). Thus, our study examined the morphology and number of neurons in the hippocampal tissue of five groups of mice and found that AD leads to overexpression of inflammatory factors and neuronal damage, with disturbed hippocampal neuronal sorting and increased numbers of apoptotic neurons observed in the DG region of AD mice, which was reversed by EX/GCA/EX+GCA. As reported in previous studies, moderate aerobic exercise and chlorogenic acid intake are beneficial for the clearance of inflammatory factors, and this was also observed in this study, with significant reductions in IL-1β, TNF-α, and IL-6, suggesting that EX and GCA could reduce inflammatory factor levels to alleviate neuroinflammation to avoid the “cytokine storm This result is most likely due to the fact that EX and GCA can reduce the levels of inflammatory factors to alleviate neuroinflammation and avoid “cytokine storm.” This result is likely to be caused by the upregulation of SIRT1 by EX or GCA, as it was previously reported that SIRT1 could mediate inhibition of the classical pro-inflammatory signaling pathway NF-kB (Davari et al., 2020; Kumar et al., 2021). Interestingly, however, when looking at the effect of the three groups EX/GCA/EX+GCA on IL-6 clearance, no significant differences were found, and there was even a tendency for the EX/EX+GCA group to have higher levels of IL-6 than GCA, which may be related to the fact that IL-6 expression is often observed to be upregulated after exercise, but according to the study, it is believed that moderate expression of IL-6 may be beneficial. Regarding the upregulation of IL-6, it may occur as a result of adaptive changes in the body to exercise. Although changes in IL-10 were not observed in our study, its anti-inflammatory effect cannot be ignored, which is one of the limitations of our study. The changes in IL-6 and IL-10 could be the paradox of exercise on the physiological functioning of the organism, with an initial adaptive up-regulation of the immune system, followed by a beneficial down-regulation of innate immunity that lasts one to two days (Pedersen, 2017; Kistner et al., 2022).

Dysregulation of the oxidative/antioxidant balance *in vivo*, leading to an oxidative stress state, is also thought to be closely associated with AD (Jiang et al., 2016; Du et al., 2018). Therefore, several oxidative stress markers were also examined in the hippocampus of mice in this study. The oxidative stress state may mediate the deposition of Aβ, associated with mitochondrial damage and reduced synaptic plasticity, which is consistent with the results of the present study, in which ROS, H2O2, and MDA levels were significantly increased in the AD brain, with ROS often thought to mediate mitochondrial damage associated with AD brain and MDA mediating lipid peroxidation in AD brain, while EX or GCA reversed this phenomenon and increased CAT. SOD and GSH-PX in order to restore the antioxidant capacity of AD mice and maintain the oxygen oxidation/antioxidant balance *in vivo*. SIRT1 may be the target responsible for this restorative effect.

In summary, Aβ deposition, oxidative stress, increased inflammatory factors, and cognitive decline were observed in AD mice in this study, but after 8 weeks of EX/GCA/EX+GCA treatment, there was a significant improvement in these phenomena. But interestingly, no significant differences were achieved between the two interventions with EX or GCA alone in most of the tested metrics, and the relief of AD The effect was almost identical, but the concomitant administration of GCA during EX showed a significant alleviation of AD relative to the other two groups.

## 5 Limitation

We must acknowledge certain limitations of our study, the first and most important being the choice of AD model, in which we chose the APP/PS1 mouse, an animal model that relies exclusively on the amyloid hypothesis, which is increasingly questioned and discredited - especially as evidence suggests that, at the clinical stage, it is tauopathy and inflammation, even though amyloidosis may be “related” to the induction of both processes (inverted commas illustrate the ambiguity and incompleteness of the understanding of these critical relationships between AD-causing proteins). The lack of translation of this classical disease model (a transgenic animal model based on the amyloid hypothesis) is now widely recognized, particularly for patients with non-familial AD and those without APOE4. However, even for patients with APOE4, it is increasingly acknowledged that therapies developed in these amyloid transgenic mouse models do not appear to be applicable to the more heterogeneous and complex clinical disease in humans, especially once human subjects have entered the clinical phase (Frisoni et al., 2022).

Secondly, regarding the lack of testing for changes in IL-10, we have only focused on changes in pro-inflammatory factors and ignored changes in anti-inflammatory factors, which are essential for terminating the inflammatory response in AD, and IL-10 is often considered one of the critical points at which exercise exerts a contributing role to the anti-inflammatory process (Islam et al., 2021).

Finally, there is less discussion about the involvement of the SIRT1 signaling pathway, where a complex mechanism is involved that regulates a number of complex signaling networks mediating physiological responses, such as the regulation of inflammation, the oxidative stress system, programmed cell death, and neurogenesis. Our study focused only on SIRT1, which may lead some inexperienced readers to believe that all of these complex changes are attributable to SIRT1; in fact, our study did not observe the role of AMPK, a possible upstream regulator of SIRT1, and the activation of AMPK by exercise/chlorogenic acid, which mediates the activation of SIRT1/PGC-1α, has been observed in most of the studies. This signaling pathway is also involved in autophagy, as previously described. The AMPK/SIRT1-mediated autophagy pathway plays an essential role in neuronal protection, with AMPK positively regulating SIRT1, and AMPKα1 overexpression activates autophagy both through activation of the AMPK/SIRT1 signaling pathway and directly (Tian et al., 2019). In addition, SIRT1 can also participate in the regulation of autophagy, but its specific mechanism needs to be confirmed by further studies. The mechanism may be related to microtubule-associated protein one light chain 3 (LC3) in the nucleus, and SIRT1 interacts with LC3 to affect the formation of autophagic vacuoles. In a mammalian study, resveratrol, a SIRT1 activator, increased the expression of LC3-II, which plays an essential role in neuroprotection by removing misfolded proteins and functionally impaired mitochondria from the cell through autophagy (Tang, 2016). Although our study observed the protective and ameliorative effects of EX+GCA in AD, readers should remain cautious when interpreting it because of the factors mentioned above.

Due to the limitation of practical conditions, the method of studying neurons in our study can only be limited to the use of HE staining, and more accurate methods, such as S100B, NeuN, or MAP2, were not used in our study. Although we also observed the loss of neurons and changes in the arrangement of hippocampal tissues, readers should interpret with caution, and it is unfortunate that the specific Number of neurons could not be observed in our study. It is unfortunate that we were unable to observe the specific number of neurons, and in the future, we hope that more specialized research methods will be used to expand and validate the relevant aspects of this study. We chose the shorter intervention time and smaller sample size used in previous studies, which may have had some impact on the results. Although our study observed a possible protective effect of EX/GCA/EX+GCA for AD and that this effect may be exerted through SIRT1/PGC-1α/BACE1, we have to admit that our study was unable to highlight a causal relationship between EX+GCA and AD, although it has been confirmed in other Mendelian randomized studies that included large sample sizes, that a causal relationship between exercise or GCA and the risk of AD, there is no study on EX+GCA, and our study is only a preliminary observation of this phenomenon, and we hope that future studies can further determine the protective effect of EX+GCA on AD by observing the causal relationship.

## 6 Conclusion

In conclusion, this study is the first to explore the effects of moderate-intensity aerobic exercise combined with chlorogenic acid administration on the APP/PS1AD model. Relative to a blank control group of wild C57BL/6 mice, the remaining groups of APP/PS1 mice all showed decreased cognitive performance, increased oxidative stress, overexpression of inflammatory factors, neuronal damage and increased Aβ plaques, but after 8 weeks of EX/GCA/EX+GCA intervention, the aforementioned conditions were effectively alleviated, and, relative to EX or GCA alone, the combination of EX+GCA showed a more meaningful beneficial effect. In turn, these remission effects may all be associated with activation of the SIRT1/PGC-1α pathway.

## Data availability statement

The original contributions presented in the study are included in the article/Supplementary material, further inquiries can be directed to the corresponding author.

## Ethics statement

The animal study was approved by Laboratory Animal Ethics Committee of Shanghai University of Traditional Chinese Medicine. The study was conducted in accordance with the local legislation and institutional requirements.

## Author contributions

DS: Conceptualization, Data curation, Methodology, Software, Validation, Visualization, Writing – original draft. ZH: Conceptualization, Methodology, Software, Validation, Visualization, Writing – original draft. WQ: Data curation, Formal analysis, Validation, Writing – original draft. FJ: Data curation, Investigation, Writing – original draft. KL: Investigation, Methodology, Visualization, Writing – original draft. XS: Data curation, Funding acquisition, Project administration, Resources, Supervision, Writing – review & editing.

## Figures and Tables

**Table 1:** 

3Rs	Reduction, Replacement, Refinement
AChE	Acetylcholinesterase
AD	Alzheimer’s disease
AMPK	Adenosine 5‘-monophosphate (AMP)-activated protein kinase
APP/PS1	(APPswe,PSEN1dE9); APP: Amyloid precursor protein
Aβ	Amyloid-β
BACE1	Beta-site amyloid precursor protein cleaving enzyme 1
BchE	Butyrylcholinesterase
CAT	Catalase from Micrococcus Lysodeikticus
CNS	Central nervous system
CON	Control group
DD H2O	Double distilled water
EX	Exercise
EX+GCA	Exercise+ Chlorogenic acid
FDA	U.S. Food and Drug Administration
GAPDH	Glyceraldehyde -3-phosphate dehydrogenase
GCA	Chlorogenic acid
GSH-PX	Glutathione peroxidase
H2O2	Hydrogen peroxide
HE	Hematoxylin-eosin staining
IL-10	Interleukin-10
IL-1β	Interleukin-1β
IL-6	Interleukin-6
LC3	MAP1LC3
MAP2	(Microtubule-associated protein) 2
MDA	Malondialdehyde
NeuN	Neuronal nuclei antigen (NeuN /RBFOX3)
OD	Optical density
PBS	Phosphate buffered saline
PGC-1α	Peroxisome proliferators-activated receptor γ coactivator l alpha
PPARγ	Peroxisome proliferator activated receptor γ
ROS	Reactive oxygen species
SIRT1	Silent Information Regulator 1
SOD	Superoxide dismutase
TNF-α	Tumor necrosis factor-α
WB	Western blot
