# Supplementary material for: Aerobic exercise combined with chlorogenic acid exerts neuroprotective effects and reverses cognitive decline in Alzheimer’s disease model mice (APP/PS1) via the SIRT1/ /PGC-1α/PPARγ signaling pathway
Source: Front Aging Neurosci. 2023 Nov 16;15:1269952. doi: 10.3389/fnagi.2023.1269952 (PMC10693339; doi:10.3389/fnagi.2023.1269952)
Supplement: Supplementary file 2 [file Data_Sheet_1.docx]

Supplementary Material

Aerobic Exercise Combined with Chlorogenic Acid Exerts Neuroprotective Effects and Reverses Cognitive Decline in Alzheimer's Disease Model Mice (APP/PS1) via The SIRT1/ /PGC-1α/PPARγ Signaling Pathway

Dan Shi, Zikang Hao, Wenxiao Qi, Fengyi Jiang, Kerui Liu and Xiao Shi

*** Correspondence:** Xiao Shi: mdshixiao@shutcm.edu.cn


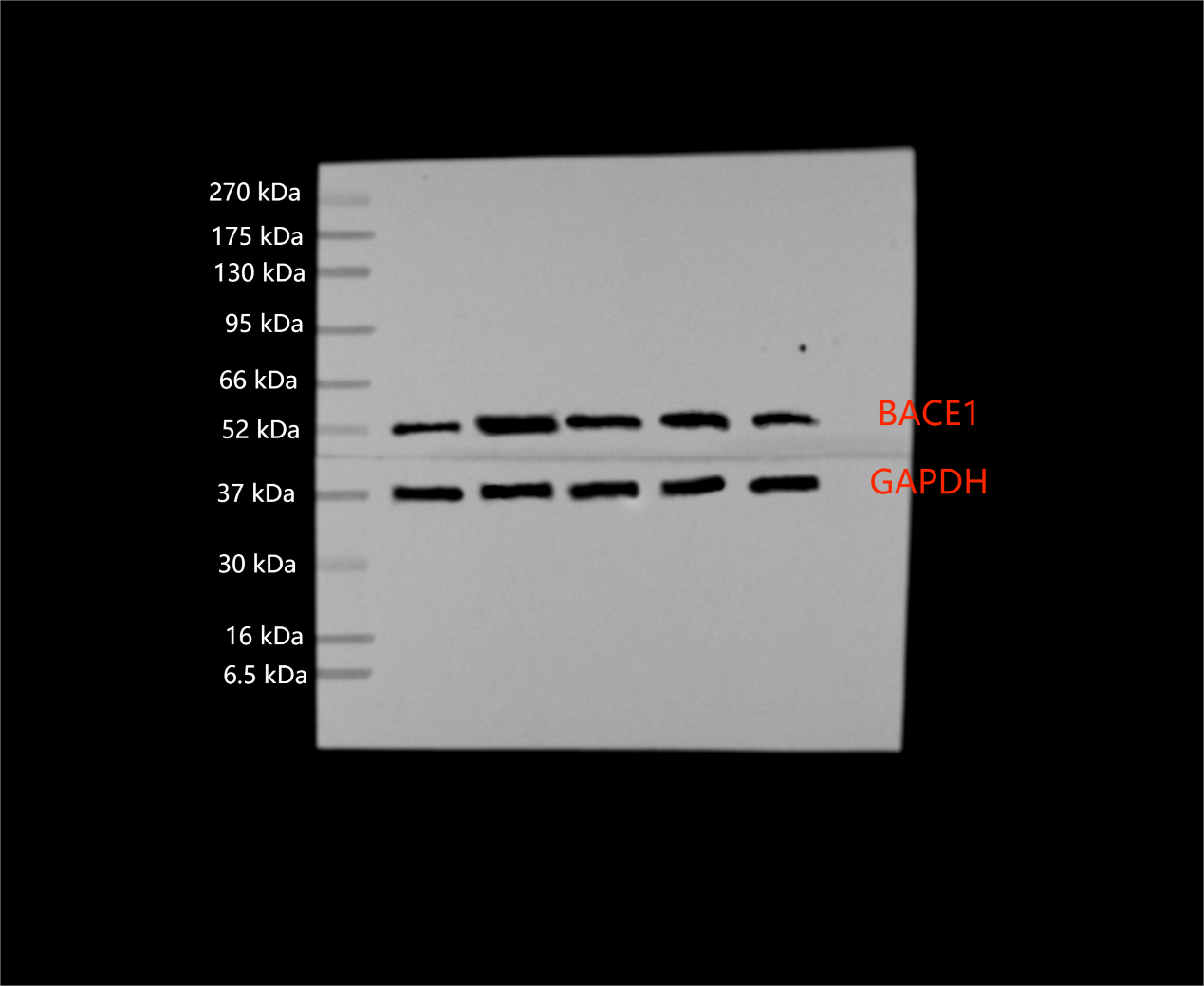
**Supplementary Figure 1.** WB raw picture of BACE1 Repeat group 1.


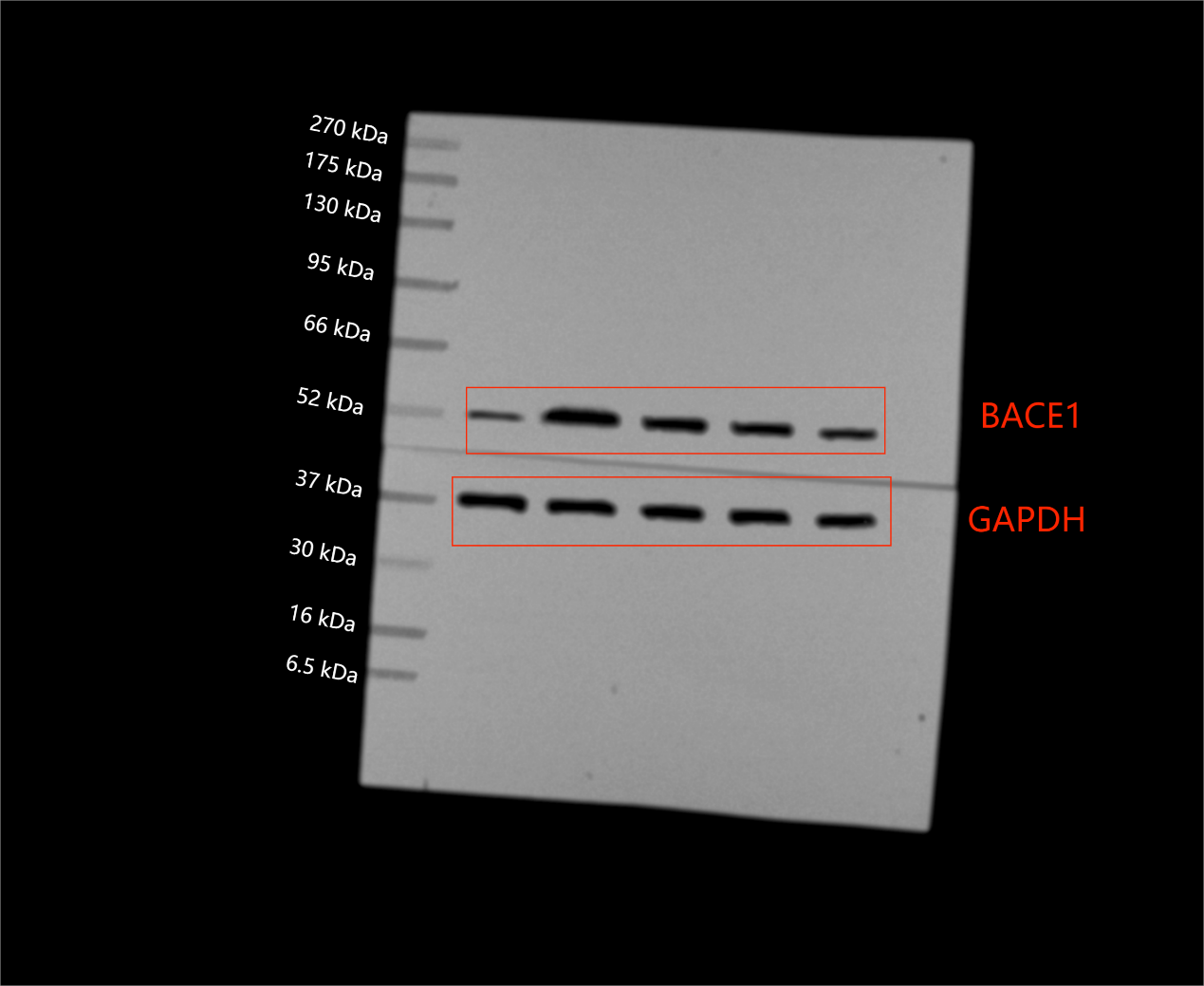


**Supplementary Figure 2.** WB raw picture of BACE1 Repeat group 2.


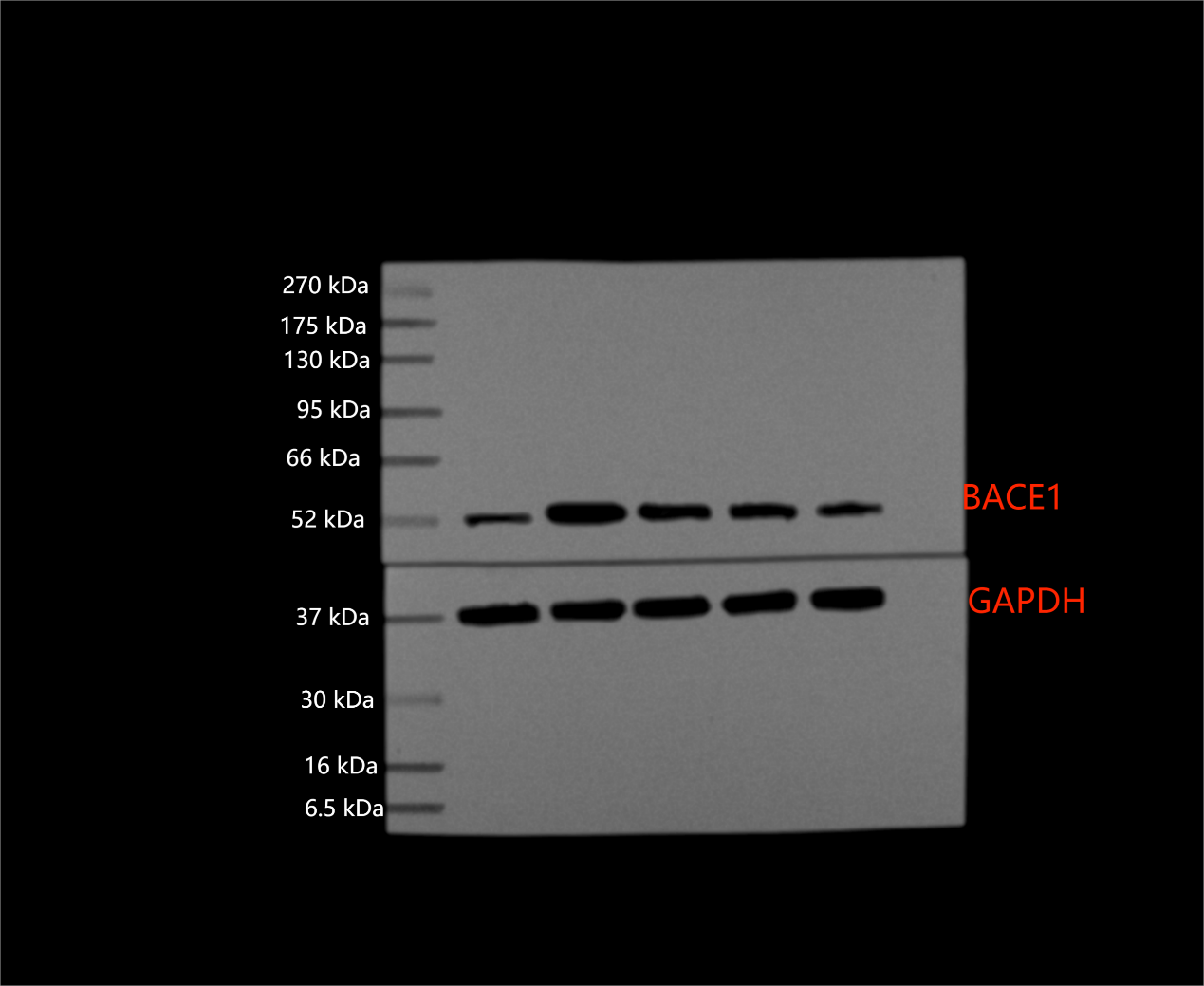


**Supplementary Figure 3.** WB raw picture of BACE1 Repeat group 3.


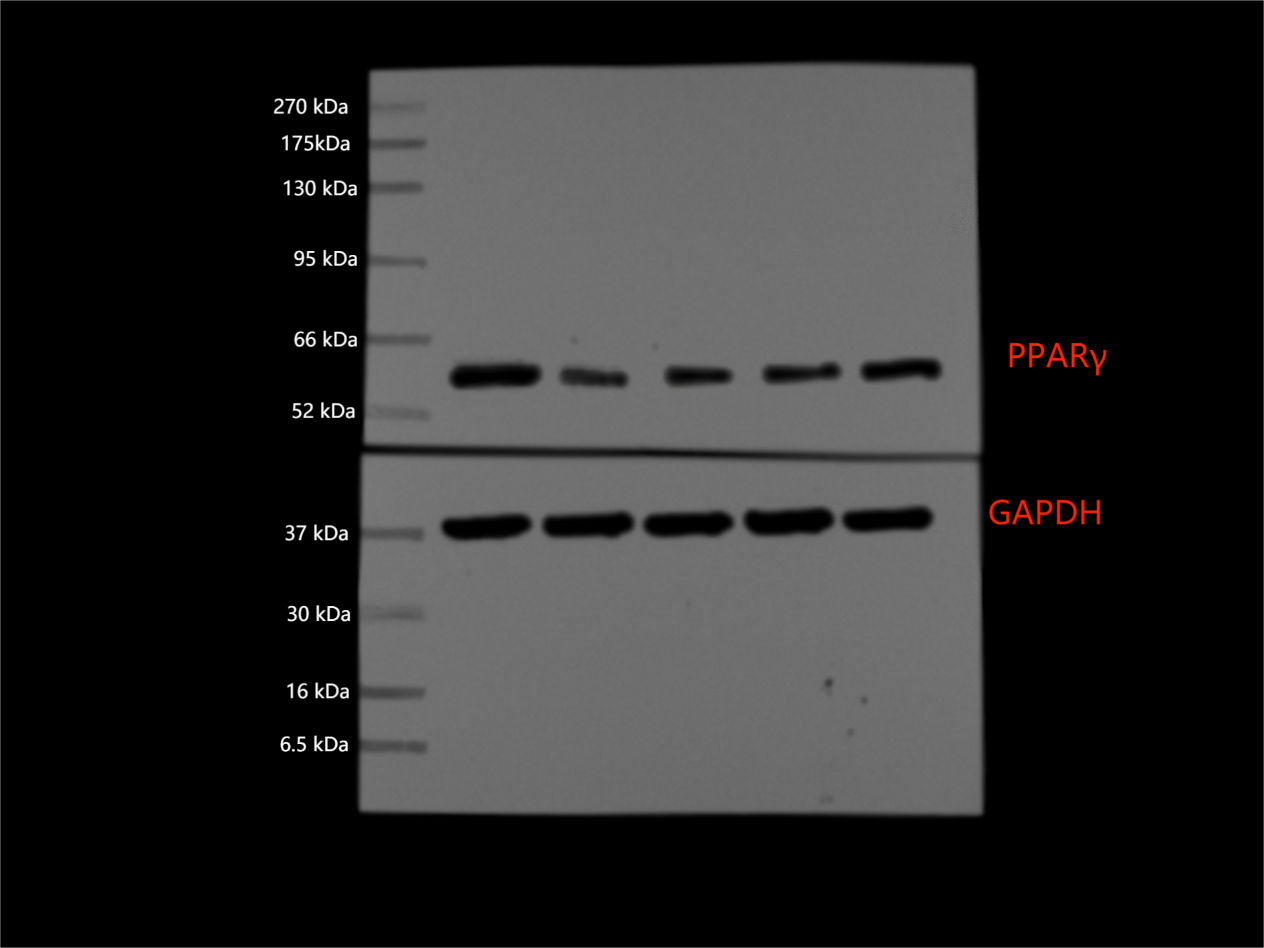


**Supplementary Figure 4.** WB raw picture of PPARγ Repeat group 1.


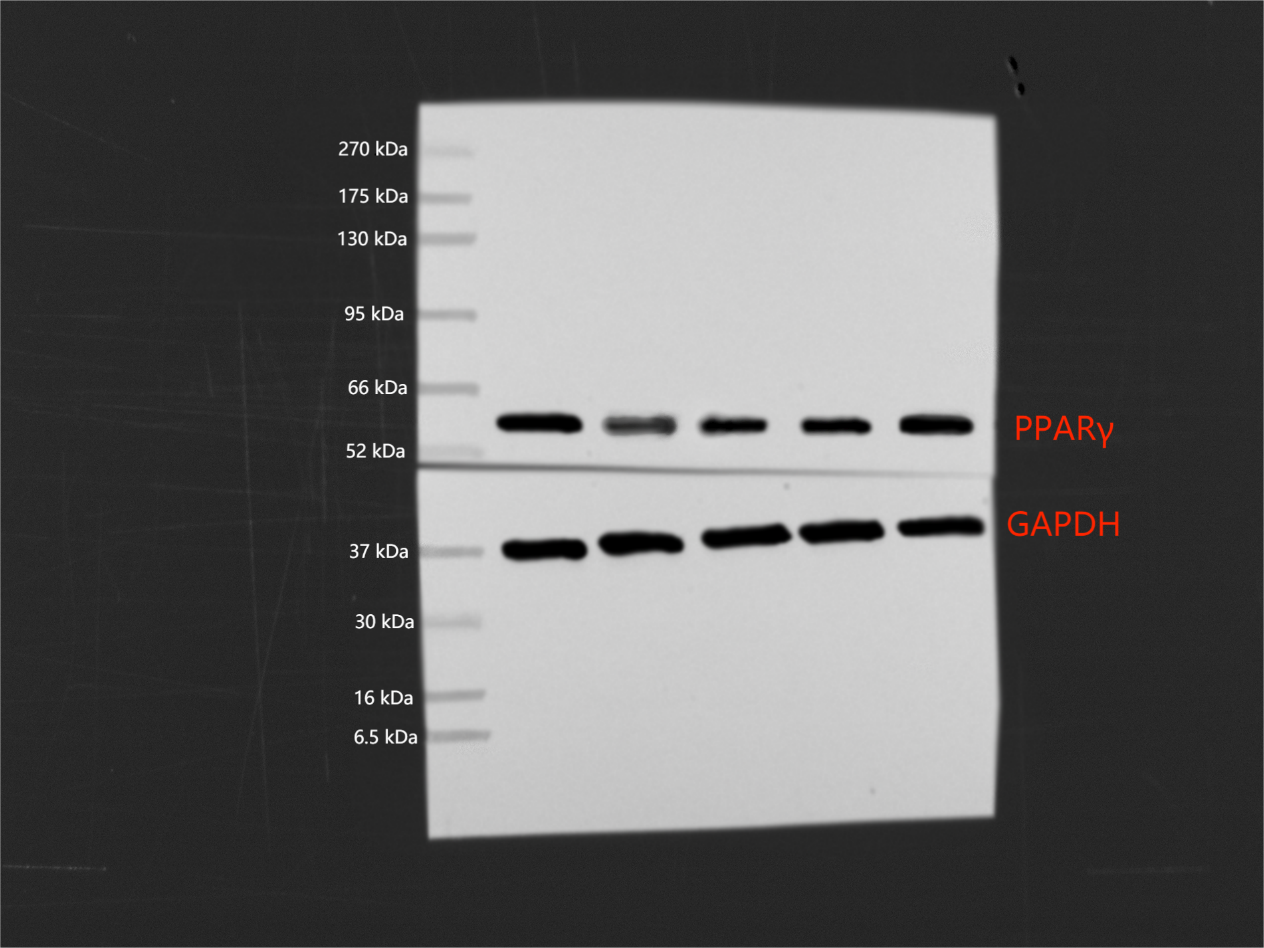


**Supplementary Figure 5.** WB raw picture of PPARγ Repeat group 2


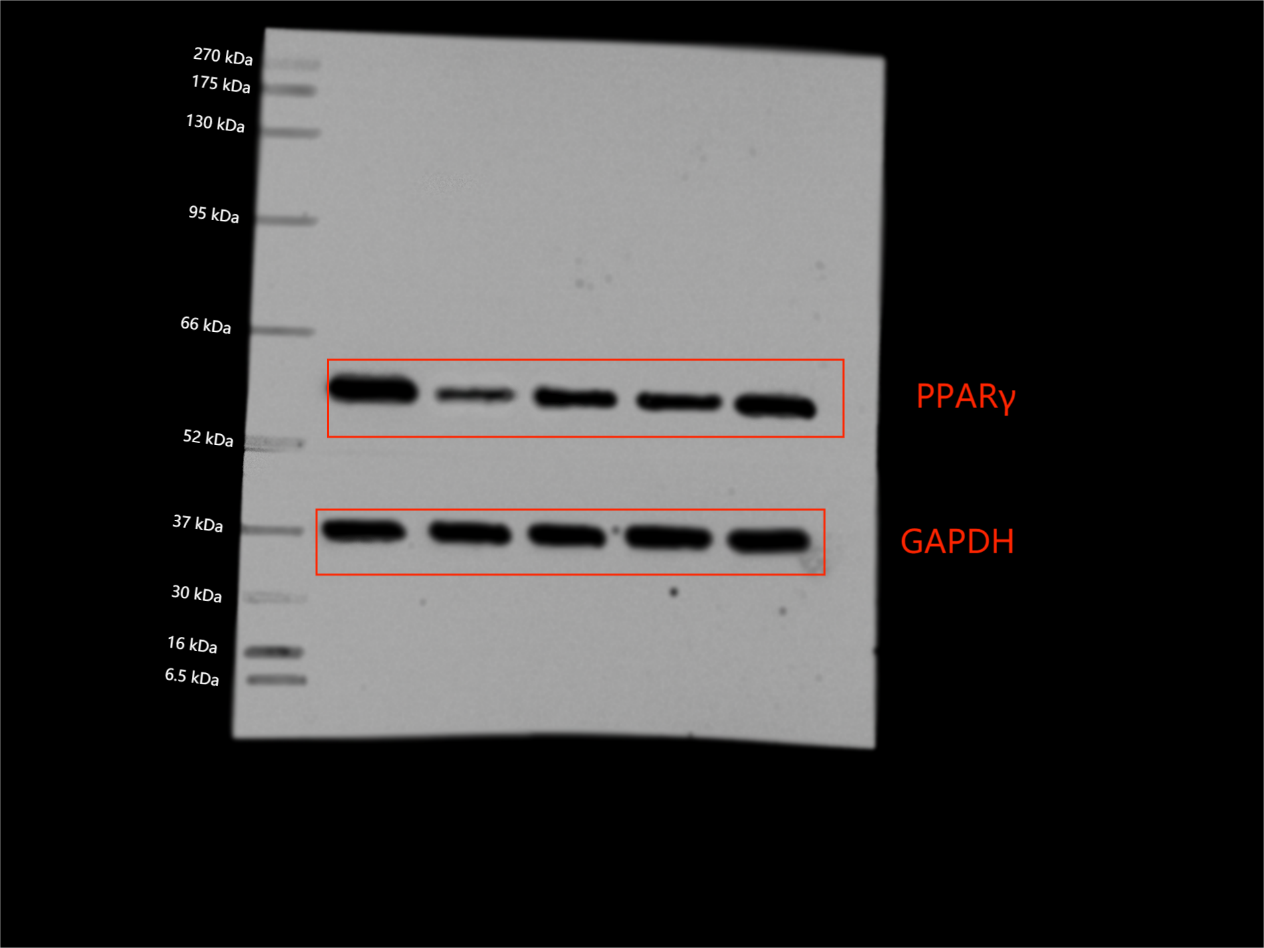
. **Supplementary Figure 6.** WB raw picture of PPARγ Repeat group 3.


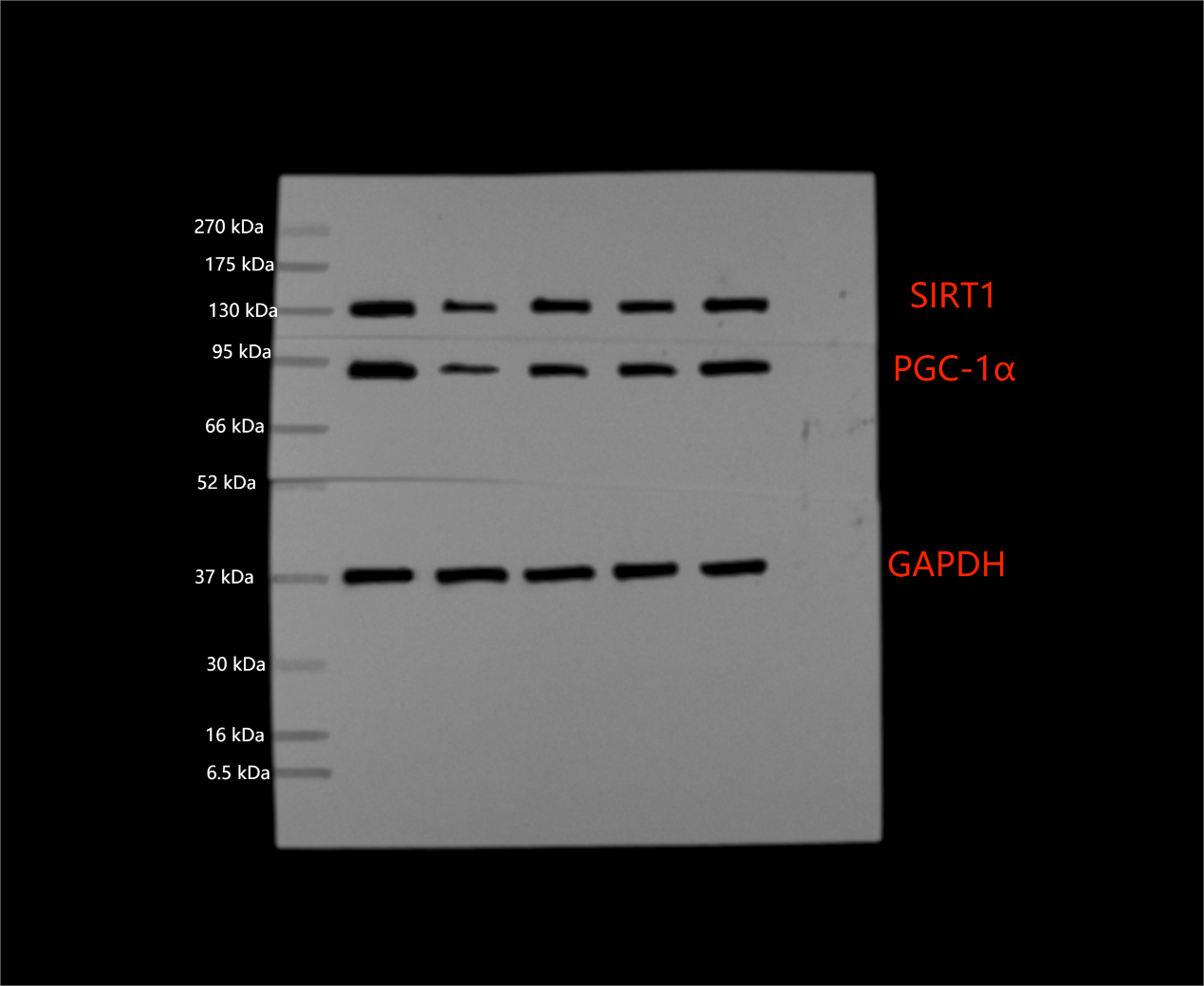
 **Supplementary Figure 7.** WB raw picture of SIRT1 and PGC-1α Repeat group 1.
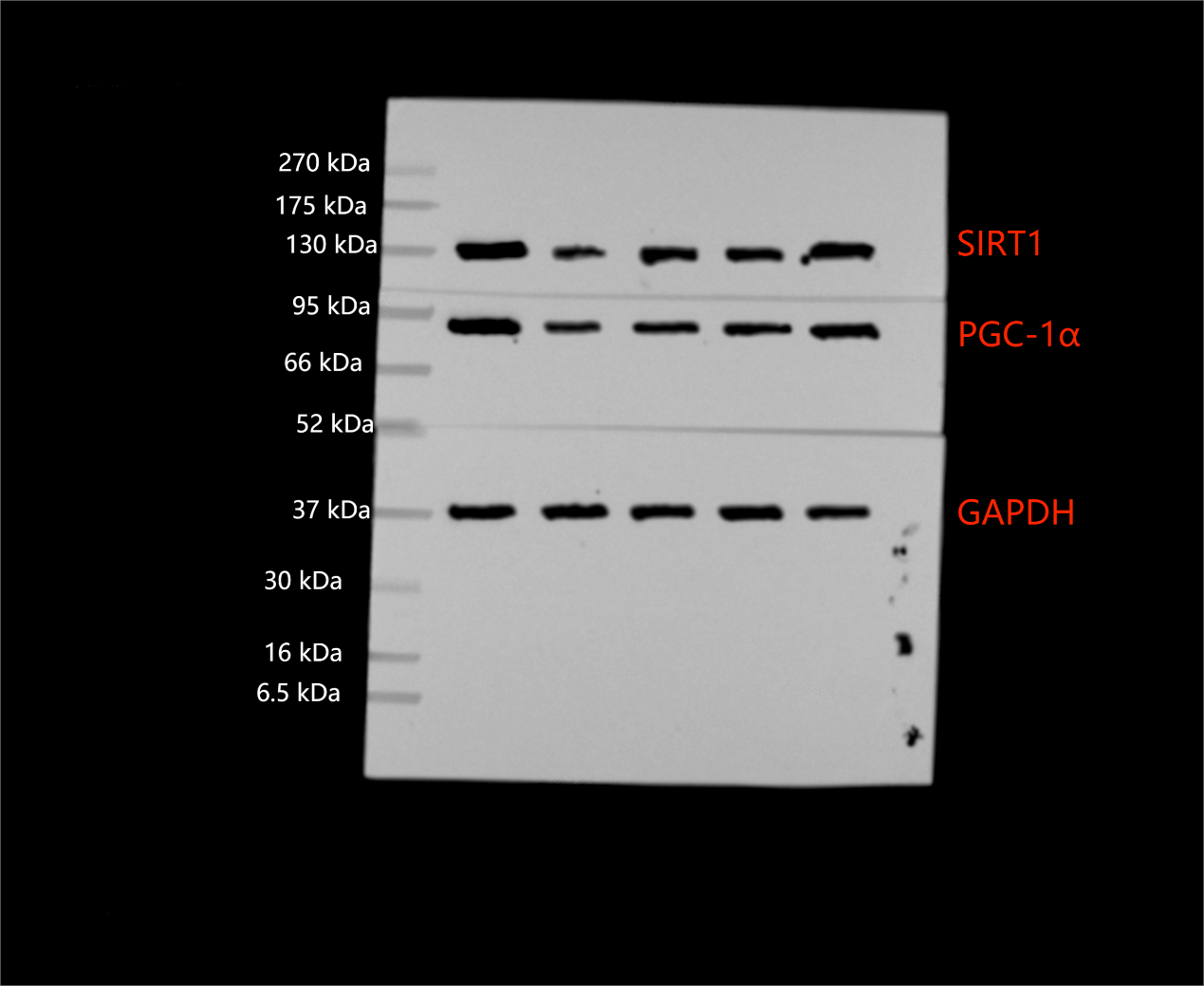
 **Supplementary Figure 8.** WB raw picture of SIRT1 and PGC-1α Repeat group 2.
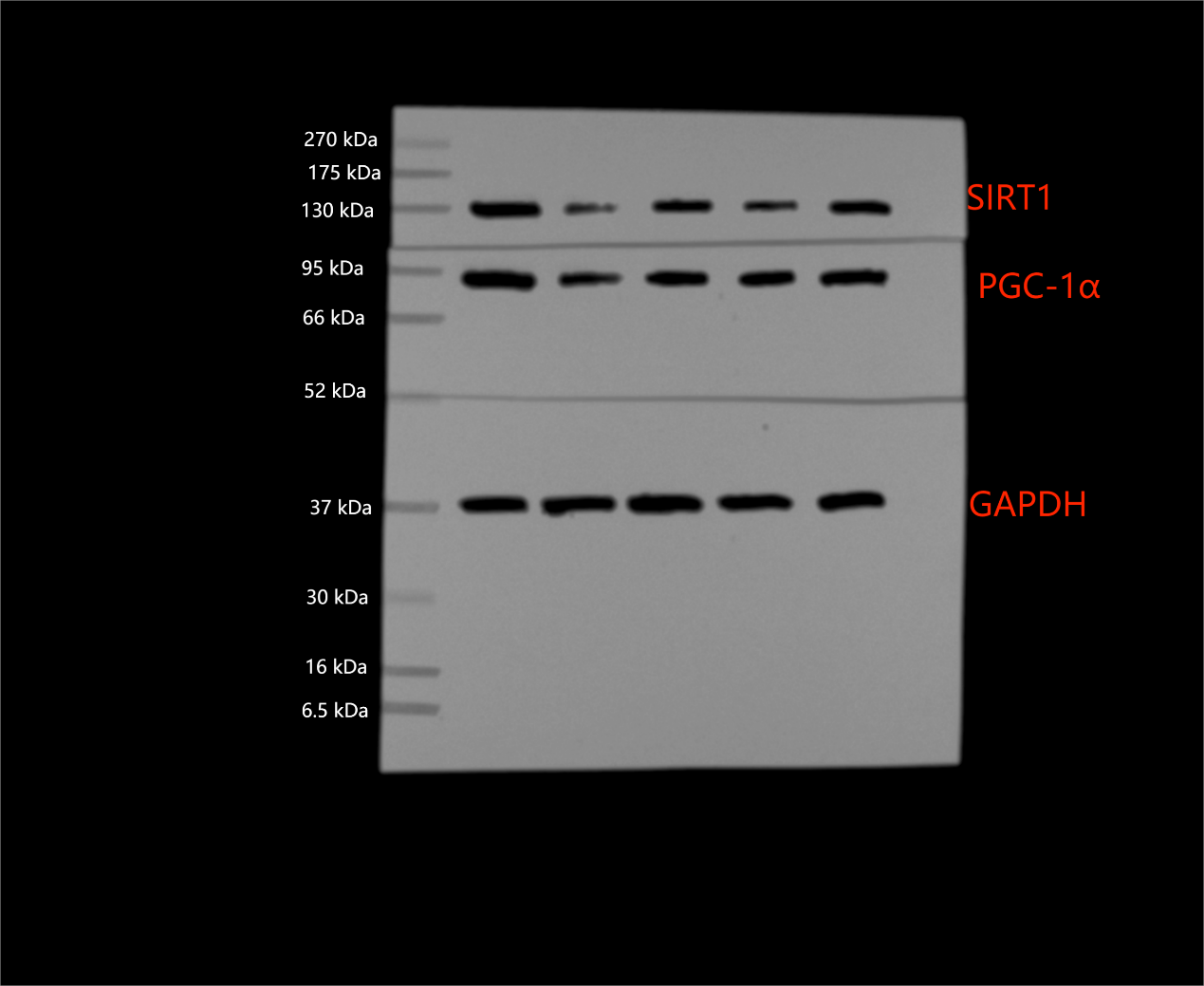


. **Supplementary Figure 9.** WB raw picture of SIRT1 and PGC-1α Repeat group 3.
